# Hypothermia Outcome Prediction after Extracorporeal Life Support for Hypothermic Cardiac Arrest Patients: Assessing the Performance of the HOPE Score in Case Reports from the Literature

**DOI:** 10.3390/ijerph182211896

**Published:** 2021-11-12

**Authors:** Nolan Grin, Valentin Rousson, Tomasz Darocha, Olivier Hugli, Pierre-Nicolas Carron, Tobias Zingg, Mathieu Pasquier

**Affiliations:** 1 School of Medicine, Lausanne University, 1011 Lausanne, Switzerland; nolan.grin@unil.ch; 2 Center for Primary Care and Public Health (Unisanté), Lausanne University Hospital, 1010 Lausanne, Switzerland; valentin.rousson@unisante.ch; 3 Severe Accidental Hypothermia Center, Department of Anaesthesiology and Intensive Care, Medical University of Silesia, 40-001 Katowice, Poland; tomekdarocha@wp.pl; 4 Department of Emergency Medicine, Lausanne University Hospital and University of Lausanne, 1011 Lausanne, Switzerland; olivier.hugli@chuv.ch (O.H.); Pierre-Nicolas.Carron@chuv.ch (P.-N.C.); 5 Department of Surgery, Lausanne University Hospital and University of Lausanne, 1011 Lausanne, Switzerland; tobias.zingg@chuv.ch

**Keywords:** cardiac arrest, ECMO, ECPR, hypothermia, accidental, potassium, publication bias, resuscitation, selection bias, triage

## Abstract

**Aims**: The hypothermia outcome prediction after extracorporeal life support (ECLS) score, or HOPE score, provides an estimate of the survival probability in hypothermic cardiac arrest patients undergoing ECLS rewarming. The aim of this study was to assess the performance of the HOPE score in case reports from the literature. **Methods**: Cases were identified through a systematic review of the literature. We included cases of hypothermic cardiac arrest patients rewarmed with ECLS and not included in the HOPE derivation and validation studies. We calculated the survival probability of each patient according to the HOPE score. **Results**: A total of 70 patients were included. Most of them (62/70 = 89%) survived. The discrimination using the HOPE score was good (Area Under the Receiver Operating Characteristic Curve = 0.78). The calibration was poor, with HOPE survival probabilities averaging 54%. Using a HOPE survival probability threshold of at least 10% as a decision criterion for rewarming a patient would have resulted in only five false positives and a single false negative, i.e., 64 (or 91%) correct decisions. **Conclusions**: In this highly selected sample, the HOPE score still had a good practical performance. The selection bias most likely explains the poor calibration found in the present study, with survivors being more often described in the literature than non-survivors. Our finding underscores the importance of working with a representative sample of patients when deriving and validating a score, as was the case in the HOPE studies that included only consecutive patients in order to minimize the risk of publication bias and lower the risk of overly optimistic outcomes.

## 1. Introduction

Accidental hypothermia is a cause of reversible cardiac arrest (CA) [1]. Patients who are successfully resuscitated with extracorporeal life support (ECLS) rewarming often have an excellent neurological outcome [2]. The hypothermia outcome prediction after ECLS (HOPE) score was derived from a retrospective cohort of 286 patients. The HOPE score provides an estimate of the probability of in-hospital survival of a given CA patient with accidental hypothermia after ECLS rewarming, based on six covariates available at hospital admission, which are age, sex, mechanism of hypothermia, core temperature (if not available, prehospital temperature), serum potassium value, and cardiopulmonary resuscitation (CPR) duration (defined as the time from initiation of CPR to the start of ECLS) [3]. An online calculator is available at www.hypothermiascore.org (accessed on 14 January 2021). The HOPE score has been externally validated in another (different) sample of 122 patients, which confirmed its good calibration and good discrimination [4]. Importantly, both studies included only consecutive ECLS patients from retrospective cohorts or hospital data selected in one determined period to minimize the risk of inclusion bias. The objective of the present study is to assess the performance of the HOPE score in non-consecutive CA patients identified through a systematic review of the literature and who had not been included in previous studies.

## 2. Methods

Published cases of non-consecutive hypothermic CA patients rewarmed with ECLS were identified through a systematic review of the literature conducted in accordance with current guidelines on systematic literature reviews, in accordance with the Preferred Reporting Items for Systematic reviews and Meta-Analyses (PRISMA) statement (Table S1) [5].

We searched the PubMed, EMBASE, Cochrane Library, and Web of Science databases without a time limit by using the keywords “hypothermia”, “cardiac arrest”, and “ECLS” and by using different Emtree and MeSH terms without language limitations. The precise research equation is presented in Table S2. The last search was performed on 30 September 2020. The search was completed by using additional cases provided by one author (M.P.) that were missed by our research strategy. One author (N.G.) conducted the literature search with the help of a professional librarian. The abstracts of the retrieved references were screened, and full texts of potentially eligible references were further examined for inclusion (NG). Duplicate patients were excluded, and the authors of case reports were contacted for supplementary data if needed. The cases for which there was any doubt concerning their eligibility were reviewed independently and blindly by another author (MP). Disagreements on eligibility were resolved by a third independent coauthor (OH). A random sample of 10% of all included cases (*n* = 7) was selected and checked by one author (MP) to verify the quality of extracted data from the articles regarding the six HOPE variables, representing 42 single data points. This verification resulted in one single discordant data (2.38%) between the two authors, consisting of a temperature difference of 0.6 °C with almost no impact on the survival HOPE score [6].

Cases of hypothermic CA after admission were included only if it occurred before ECLS. Patients in CA with a temperature of > 32 °C were excluded, assuming that CA was not induced by hypothermia in these cases. We excluded patients who were not in CA when ECLS was started, those with missing HOPE variables, and those rewarmed with another technique than ECLS (peritoneal lavage, thoracic lavage, or hemodialysis), as well as those included in our previous derivation and validation of the HOPE score [3,4].

We added 30 min to start the extracorporeal membrane oxygenation for two patients, as the specific time interval between hospital admission and beginning of ECLS was not mentioned. The mechanisms of hypothermia were classified as non-asphyxia-related (e.g., outdoor or indoor exposure to cold, immersion) or asphyxia-related (e.g., submersion, avalanche with burial of the head under the snow and in CA at extrication). As in the previous studies [3,4], the primary outcome was survival to hospital discharge. The neurological outcome at hospital discharge was assessed for the survivors by the cerebral performance category (CPC) [3,4]. A CPC of 1 or 2 was considered as favorable [3,4].

The HOPE score for each patient was calculated as follows (Equation (1)),

(1) $$\mathrm{score}=2.44-1.55\times \mathrm{male}-1.95\times (\mathrm{asphyxia}-\mathrm{related}\;\mathrm{mechanism})\;-0.0191\times \mathrm{age}-2.07\times \log_{2}\;\mathrm{potassium}-0.573\times \log_{2}\;\left(\mathrm{CPR}\;\mathrm{duration}\right)\;+0.937\times \mathrm{temperature}\;\mathrm{Celsius}-0.0247\times {\mathrm{temperature}\;\mathrm{Celsius}}^{2}$$

The HOPE survival probability was then obtained as (Equation (2))

The HOPE survival probability = exp(score)/(1 + exp(score))(2)

Sensibility, specificity, positive, and negative predictive values of the HOPE score were evaluated, applying a threshold of 10% to the HOPE survival probability (i.e., those patients with a HOPE survival probability ≥10% were identified as survivors), and the threshold suggested to initiate ECLS rewarming [3,4].

As only previously published and anonymous data were used, our study was exempted from formal ethical approval.

### Statistical Analysis

Descriptive statistics were expressed as frequencies for categorical data, and medians and interquartile ranges for continuous data. Survivors and non-survivors were compared using a Fisher’s exact test for categorical data and a Mann–Whitney test for continuous data. Confidence intervals (CI) for proportions have been calculated according to the Wilson method. Data were retrieved from the patient information database that was established for this study and were exported into Stata version 14 (Stata Corporation, College Station, TX, USA) for analysis. Confidence intervals for the area under the curve of the receiver operating characteristic (in what follows, AUC) were calculated via the *ci.auc* function available in the *pROC* library from the R statistical software version 3.3 (R Core Team, Vienna, Austria).

## 3. Results

**Figure 1:**
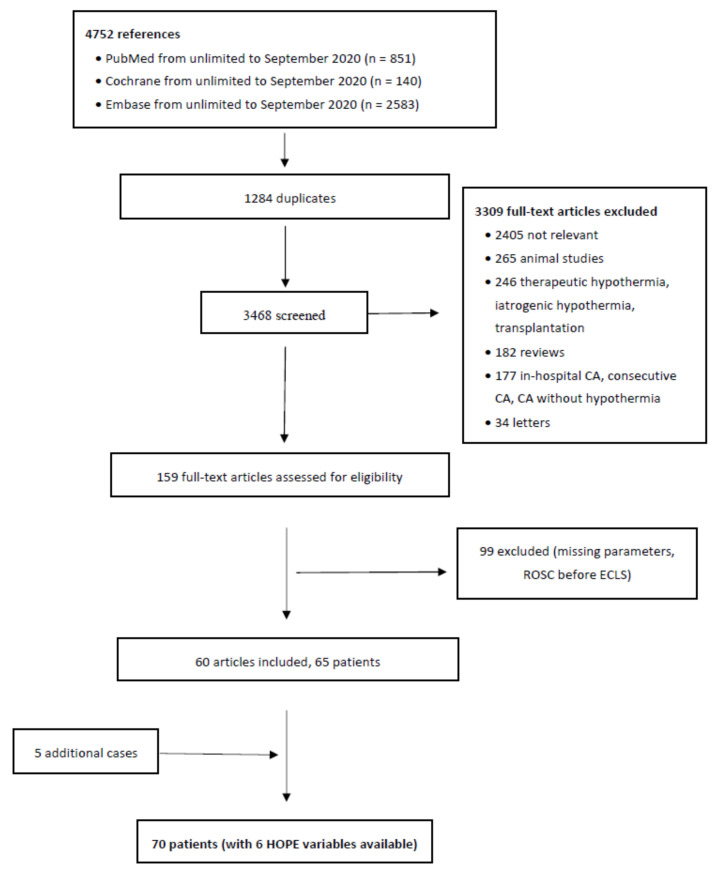
Flow chart for patient selection in the systematic review of patients with hypothermic cardiac arrest. The five additional cases are case reports from the literature provided by one author and that were missed by our research strategy. CA = cardiac arrest; ECLS = extracorporeal life support; HOPE = hypothermia outcome prediction after ECLS; ROSC = return of spontaneous circulation.

The research strategy retrieved 70 patients for whom the six HOPE variables were available (Figure 1, Table S3).

Patients’ clinical characteristics and HOPE variables are summarized in Table 1. A total of 62 (89%) patients survived, and 8 (11%) patients died. The CPC category was available for all (100%) of the survivors and was favorable (i.e., CPC 1 or 2) for 58 (94%) of them, and it was unfavorable (CPC 3) for 4 (5.7%).

**Figure 2:**
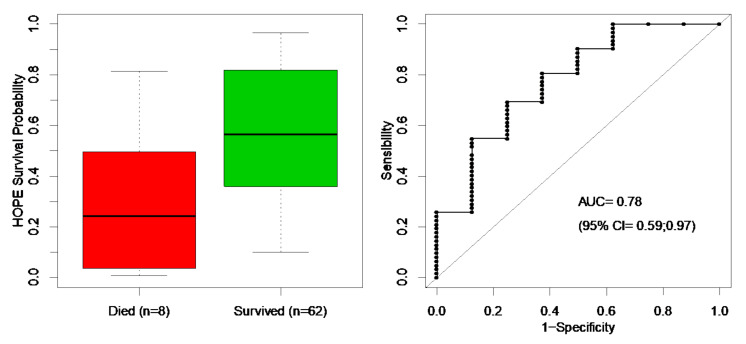
Hypothermia outcome prediction after extracorporeal life support (HOPE) survival probabilities (**left panel**) and receiver operating characteristic curve (**right panel**) of the survival probabilities estimated from 70 patients. The average HOPE survival probability was 57% for survivors (in green) and 30% for non-survivors (in red). AUC = area under the curve; CI = confidence interval.

The distribution of HOPE survival probabilities ranged from 0.7% to 96.5%, averaging 54%. The distribution of HOPE survival probabilities differed markedly between survivors and non-survivors, averaging 57% for the former and 30% for the latter (*p* < 0.01); the HOPE score achieved an AUC of 0.78 (95% CI: 0.59–0.97), suggesting good discrimination (Figure 2).

The HOPE survival probabilities for the eight non-survivors were 0.7%, 3.4%, 3.6%, 14.7%, 33.6%, 43.9%, 55.3%, and 81.4%. Using a HOPE survival probability of at least 10% as criterion to decide to rewarm a patient, we would have a specificity of 3/8 = 38%, which would represent 5 (7%) false positives (futile rewarming). On the other hand, all survivors had a HOPE survival probability higher than 10%, except for one who achieved 9.9%, yielding a sensitivity of 61/62 = 98% and a single false negative. Sensitivity, specificity, positive, and negative predictive values, together with 95% CI, are provided in Table 2. The positive predictive value in the present study was high (92% compared to less than 60% in the HOPE derivation and validation studies) due to the over-representation of survivors in case reports.

## 4. Discussion

In this study, we evaluated the performance of the HOPE score on a sample of 70 non-consecutive patients who were described in case reports from the literature. While the calibration of the HOPE score was rather poor in this sample, as it predicted 54% survivors when 89% survivors were observed, its discrimination remained good with an AUC close to 0.8. Using a HOPE survival probability threshold of at least 10% as a decision criterion for rewarming a patient, as has been suggested in the HOPE derivation and validation studies, would result in only five false positives (futile rewarming) and a single false negative, leaving us with 64 (or 91%) correct decisions, which can certainly be considered as a good practical performance.

The HOPE score has been derived and externally validated on two (different) samples of consecutive patients. All three studies—the HOPE derivation and validation studies, as well as the present study—demonstrated good discrimination of the HOPE score between survivors and non-survivors. The AUC in the present study (0.78, 95% CI: 0.59–0.97) was just a bit lower than in the HOPE validation study (0.825, 95% CI: 0.75–0.90), although the confidence intervals are largely overlapping. The fact that the confidence interval was larger for the present study than in the HOPE validation study was due to its smaller sample size (*n* = 70 vs. *n* = 122). The positive predictive value in the present study was high in the present study due to the over-representation of survivors in case reports.

In the HOPE validation study, not only the discrimination but also the calibration of the HOPE score was found to be good. Over the 70 patients included in the present study, the average of the HOPE survival probabilities was 54%, which is well and significantly below the observed proportion of 89% of survivors. In other words, we had in this sample of non-consecutive patients too many survivors compared to what would be expected using the HOPE score, indicating that the HOPE score was not well calibrated for such patients. That the calibration of the HOPE score was poor in the present study was clearly due to an expected selection bias. It has been shown that studies with significant results are more likely to be published, and papers with positive results have a greater chance of being published in higher impact journals [7]. Publication bias has been shown to be more frequently present because of the author’s preference than because of the journal’s preference [8]. Lack of positive/significant results is cited as the first reason of failure to finalize and publish an article [7], as authors spend more time on studies with good and relevant outcomes, they are reluctant to waste time on something without apparent impact. Cases with unfavorable outcomes are therefore less likely to be published, and, even when accepted, the publication of articles with negative outcomes are delayed, which can in turn influence the results and conclusions of associated research such as meta-analyses [9,10].

The survival rate in the present study was 89%, which is much higher than in the HOPE derivation (37%) and validation (42%) studies, which included only consecutive cases [3,4]. This supports the hypothesis of publication bias, as also reported by others [11]. In a meta-analysis of randomized and observational studies of patients with accidental hypothermia who required ECLS, the survival rate of the entire cohort (658 patients) was 46%, but it was as high as 95% (*n* = 38) for the 40 case reports [11]. One may have expected that patients selected from large case series or retrospective studies originating from centers with a high caseload and experience would have better outcomes than those from smaller centers. However, the opposite was the case, which is likely explained by selection bias, in other words, cases with bad outcomes are underrepresented. This finding supports the methodology used in HOPE studies that included only consecutive patients in order to minimize the risk of publication bias [12]. This approach resulted in lower, and probably more realistic, survival rates than in the present study.

All but one of the survivors had a HOPE-estimated survival probability higher than 10%. The detection of all (or at least most) potentially surviving hypothermic CA patients is critical because of the good prognosis and neurological outcome in comparison with normothermic heart arrest [2]. The only false negative, i.e., the only patient who survived with a HOPE survival probability lower than 10%, was a 6-year-old boy with a HOPE survival probability of 9.92% (which would have actually been rounded to 10% using the online calculator) [3,13]. He was submerged in water with a core temperature of 17 °C and a potassium level of 7.6 mmol/L at hospital admission. ECLS with cardiopulmonary bypass started after 95 min of CPR, and the boy completely recovered. It might be useful to recall that the HOPE score must be correlated with the clinical scenario. Borderline values (scores near the cut-off of 10%) must be discussed in terms of the overall situation of the patient, especially for children, who will obviously live longer than adults if they manage to survive. In children, the latest guidelines actually recommend seeking expert consultation rather than using the HOPE score to guide rewarming decisions [14]. HOPE should not be considered a substitute for clinical judgment or assessment.

It might also be useful here to recall that if the situation and the scenario are not entirely clear, the more favorable option for the patient is to be chosen. For example, the present study included a 44-year-old woman for whom hypothermia by immersion or submersion in water was uncertain [15]. Depending on which clinical scenario is selected in the score, the probability of survival changes from 16% with submersion to 57% with immersion. In this example, both values are higher than the cut-off, but the magnitude of the difference demonstrates that, when in doubt, it is important to choose the variant leading to the patient’s highest survival probability. Rewarming with ECLS can be an option for those patients with a survival probability near the 10% cut-off value or in an unclear situation with any doubt about the events.

### Limitations

The most important limitation of this study is the low number of non-survivors and our subsequent low ability to draw conclusions about the negative predictive value of HOPE at a 10% cut-off value. The proportion of patients with a HOPE score of <10% was also low (6%) compared to the HOPE derivation (31%) and validation (29%) studies [3,4]. These limitations are, however, the direct consequence of publication bias that we expected to find in the study.

## 5. Conclusions

In this highly selected sample, the HOPE score still had a good practical performance in terms of discrimination, false positives, and false negatives rates. The selection bias most likely explains the poor calibration found in the present study. Our finding underscores the importance of working with a representative sample of patients when deriving and validating a score, and it supports the methodology used in HOPE studies that included only consecutive patients in order to minimize the risk of publication bias and lower the risk of overly optimistic outcomes.

## Figures and Tables

**Table 1:** Patient characteristics and outcomes (*n* = 70).

	Overall (*n* = 70)	Survivors 62 (89%)	Non-Survivors 8 (11%)	*p* Value
**Continuous variables, median (IQR)**				
Age (years)	33 (1–95)	39.5 (1–95)	10.5 (2–65)	0.02
Temperature (°C)	22.7 (13.5–28.9)	23 (13.5–28.9)	22 (20.7–25)	0.70
Potassium (mmol/L)	4.2 (2.4–16.1)	4.2 (2.4–11.8)	5.8 (2.9–16.1)	0.16
CPR duration (min)	95 (20–307)	95 (20–307)	125 (60–263)	0.12
pH (60/70 patients)	7.0 (6.4–7.8)	7.0 (6.4– 7.8)	6.8 (6.4–7.3)	0.28
Lactate (mmol/L) (30/70 patients)	13 (3–31)	13 (3–25)	31 (31–31)	0.09
PaCO_2_ (kPa) (41/70 patients)	7.33 (2.48–29.73)	6.3 (2.48–19.1)	10.38 (7.3–29.73)	0.08
**Categorical variables, n (%)**				
Sex				0.19
Female	23 (33%)	22 (96%)	1 (4%)	
Male	47 (67%)	40 (85%)	7 (15%)	
Mechanism				0.02
Exposure	41 (59%)	39 (95%)	2 (5%)	
Immersion	9 (13%)	9 (100%)	0 (0%)	
Submersion	15 (21%)	10 (67%)	5 (33%)	
Avalanche	5 (7%)	4 (80%)	1 (20%)	
Asphyxia-related mechanism				0.002
Yes ^a^	20 (29%)	14 (70%)	6 (30%)	
No ^b^	50 (71%)	48 (96%)	2 (4%)	
Cardiac rhythm				0.20
Asystole	36 (54%)	30 (83%)	6 (17%)	
Ventricular fibrillation	28 (42%)	27 (96%)	1 (4%)	
PEA	3 (4%)	3 (100%)	0 (0%)	
CA circumstance				0.09
Unwitnessed CA	52 (75%)	44 (85%)	8 (15%)	
Witnessed CA	17 (25%)	17 (100%)	0 (0%)	
Type of ECLS				0.25
CPB	49 (70%)	42 (86%)	7 (14%)	
ECMO	21 (30%)	20 (95%)	1 (5%)	

^a^ Submersion, avalanche with burial of the head under the snow; ^b^ Outdoor or indoor exposure to cold, immersion. CA = cardiac arrest; CPB = cardiopulmonary bypass; CPR = cardiopulmonary resuscitation; ECLS = extracorporeal life support; ECMO = extracorporeal membrane oxygenation; IQR = interquartile range; PEA = pulseless electrical activity.

**Table 2:** Diagnostic performance when using HOPE survival probability ≥10% as criterion to decide to rewarm a patient (CI = confidence interval; HOPE = hypothermia outcome prediction after extracorporeal life support).

	Sensitivity ^a^	Specificity ^b^	PPV ^c^	NPV ^d^	FP ^e^	FN ^e^
**HOPE derivation study (*n* = 286) ^f^**						
HOPE ≥ 10%	106/106 = 100%	92/180 = 51%	106/194 = 55%	92/92 = 100%	88/286 = 31%	0/286 = 0%
(95% CI)	(97–100%)	(44–58%)	(48–61%)	(96–100%)	(26–36%)	(0–1%)
**HOPE validation study (*n* = 122) ^f^**						
HOPE ≥ 10%	50/51 = 98%	34/71 = 48%	50/87 = 57%	34/35 = 97%	37/122 = 30%	1/122 = 1%
(95% CI)	(90–100%)	(37–59%)	(47–67%)	(85–99%)	(85–99%)	(0–4%)
**Present study (*n* = 70)**						
HOPE ≥ 10%	61/62 = 98%	3/8 = 38%	61/66 = 92%	3/4 = 75%	5/70 = 7%	1/70 = 1%
(95% CI)	(91–100%)	(14–69%)	(83–97%)	(30–95%)	(3–16%)	(0–8%)

^a^ Sensitivity is defined as the probability that the criterion is fulfilled among the survivors. ^b^ Specificity is defined as the probability that the criterion is not fulfilled among the non-survivors. ^c^ The positive predictive value (PPV) is defined as the proportion of patients who survived among those fulfilling the criterion. ^d^ The negative predictive value (NPV) is defined as the proportion of patients who died among those not fulfilling the criterion. ^e^ FP denotes the percentage of false positive and FN the percentage of false negative results (calculated over all patients, whether positive or negative). ^f^ Pasquier M, Rousson V, Darocha T, et al. Hypothermia outcome prediction after extracorporeal life support for hypothermic cardiac arrest patients: An external validation of the HOPE score. Resuscitation. 2019;139:321–8.

## Data Availability

The data may be available on reasonable request to the corresponding author.

## Supplementary Materials

The following are available online at https://www.mdpi.com/article/10.3390/ijerph182211896/s1, Table S1: Prisma checklist, Table S2: Detailed research methodology, Table S3: Source and characteristics of the 70 selected patients identified through the literature review (*n* = 64 references).
