# Supplementary material for: Hypothermia Outcome Prediction after Extracorporeal Life Support for Hypothermic Cardiac Arrest Patients: Assessing the Performance of the HOPE Score in Case Reports from the Literature
Source: Int J Environ Res Public Health. 2021 Nov 12;18(22):11896. doi: 10.3390/ijerph182211896 (PMC8622062; doi:10.3390/ijerph182211896)
Supplement: Supplementary file 1 [file ijerph-18-11896-s001.zip › ijerph-1389347-supplementary.pdf]

International Journal of
Environmental Research and Public Health

MDPI

Supplementary file

Table S1. Prisma checklist.

Section/Topic	#	Checklist Item	Reported on Page #
	TITLE		
Title	1	Identify the report as a systematic review, meta-analysis, or both.	1
	ABSTRACT		
Structured summary	2	Provide a structured summary including, an applicable: background; objectives; data sources; study eligibility criteria, participants, and interventions; study appraisal and synthesis methods; results; limitations; conclusions and implications of key findings; systematic review registration number.	2
	INTRODUCTION		
Rationale	3	Describe the rationale for the review in the context of what is already known.	3
Objectives	4	Provide an explicit statement of questions being addressed with reference to participants, interventions, comparisons, outcomes, and study design (PICOS).	3
	METHODS		
Protocol and registration	5	Indicate if a review protocol exists, if and where it can be accessed (e.g., Web address), and, if available, provide registration information including registration number.	
Eligibility criteria	6	Specify study characteristics (e.g., PICOS, length of follow-up) and report characteristics (e.g., years considered, language, publication status) used as criteria for eligibility, giving rationale.	4
Information sources	7	Describe all information sources (e.g., databases with dates of coverage, contact with study authors to identify additional studies) in the search and date last searched.	4
Search	8	Present full electronic search strategy for at least one database, including any limits used, such that it could be repeated.	4

Study selection	9	State the process for selecting studies (i.e., screening, eligibility, included in systematic review, and, if applicable, included in the meta-analysis).	4
Data collection process	10	Describe method of data extraction from reports (e.g., piloted forms, independently, in duplicate) and any processes for obtaining and confirming data from investigators.	4
Data items	11	List and define all variables for which data were sought (e.g., PICOS, funding sources) and any assumptions and simplifications made.	4
Risk of bias in individual studies	12	Describe methods used for assessing risk of bias of individual studies (including specification of whether this was done at the study or outcome level), and how this information is to be used in any data synthesis.	
Summary measures	13	State the principal summary measures (e.g., risk ratio, difference in means).	5
Synthesis of results	14	Describe the methods of handling data and combining results of studies, if done, include measures of consistency (e.g., I^2) for each meta-analysis.	5
Risk of bias across studies	15	Specify any assessment of risk of bias that may affect the cumulative evidence (e.g., publication bias, selective reporting within studies).	
Additional analyses	16	Describe methods of additional analyses (e.g., sensitivity or subgroup analyses, meta-regression), if done, indicate which were pre-specified.	5
	**RESULTS**		
Study selection	17	Give numbers of studies that were screened, assessed for eligibility, and included in the review, with reasons for exclusions at each stage, ideally with a flow diagram.	6
Study characteristics	18	For each study, present characteristics for which data were extracted (e.g., study size, PICOS, follow-up period) and provide the citations.	6
Risk of bias within studies	19	Present data on risk of bias of each study and, if available, any	6

		outcome level assessment (see item 12).	
Results of individual studies	20	For all outcomes considered (benefits or harms), present, for each study, (a) simple summary data for each intervention group (b) effect estimates and confidence intervals, ideally with a forest plot.	6
Synthesis of results	21	Present results of each meta-analysis done, including confidence intervals and measures of consistency.	6
Risk of bias across studies	22	Present results of any assessment of risk of bias across studies (see Item 15).	
Additional analysis	23	Give results of additional analyses, if conducted (e.g., sensitivity or subgroup analyses, meta-regression [see Item 16]).	6
	**DISCUSSION**		
Summary of evidence	24	Summarize the main findings including the strength of evidence for each main outcome; consider their relevance to key groups (e.g., healthcare providers, users, policy makers).	7–8–9
Limitations	25	Discuss limitations at study and outcome level (e.g., risk of bias) and at review-level (e.g., incomplete retrieval of identified research, reporting bias).	7–8–9
Conclusions	26	Provide a general interpretation of the results in the context of other evidence and implications for future research.	7–8–9
	**FUNDING**		
Funding	27	Describe sources of funding for the systematic review and other support (e.g., supply of data); this is the role of funders for the systematic review.	10–11–12

From: Moher D, Liberati A, Tetzlaff J, Altman DG, The PRISMA Group (2009). Preferred Reporting Items for Systematic Reviews and Meta-Analyses: The PRISMA Statement. PLoS Med 6(7): e1000097. doi:10.1371/journal.pmed1000097. For more information, visit: www.prisma-statement.org (accessed on 8 April 2019).

Table S2. Detailed research methodology.

Concepts	Hypothermia	Heart Arrest	ECLS
**Free words**	Rewarming<br>Hypothermi*<br>Body temperature change	Heart arrest<br>Cardiac arrest<br>Cardiopulmonary Arrest<br>Asystole<br>Cardiopulmonary resuscitation	Extracorporeal*<br>ECMO<br>ECLS<br>cardiopulmonary bypass
**Emtree**	'hypothermia'/exp<br>'warming'/exp	'heart arrest'/exp<br>'cardiopulmonary arrest'/exp	'extracorporeal circulation'/exp<br>'extracorporeal oxygenation'/exp
**MeSH**	"Rewarming"[Mesh] OR "Hypothermia"[Mesh]	"Heart Arrest"[Mesh] OR "Cardiopulmonary Resuscitation" [Mesh]	"Extracorporeal Circulation" [Mesh]

Pubmed 30 September 2020

Equation:

("Rewarming"[Mesh] OR "Hypothermia"[Mesh] OR Rewarming[tiab] OR Hypothermi*[tiab] OR Body temperature change*[tiab]) AND ("Heart Arrest"[Mesh] OR "Cardiopulmonary Resuscitation"[Mesh] OR Heart arrest*[tiab] OR Cardiac arrest*[tiab] OR Cardiopulmonary Arrest*[tiab] OR Asystole*[tiab] OR Cardiopulmonary resuscitation*[tiab]) AND ("Extracorporeal Circulation"[Mesh] OR Extracorporeal*[tiab] OR ECMO[tiab] OR ECLS[tiab] OR cardiopulmonary bypass[tiab]).

Cochrane 30 September 2020

Equation:

((rewarming OR hypothermi* OR (body NEAR/3 temperature NEAR/3 change*)):ab,ti,kw) AND ((((heart OR cardiac OR cardiopulmonary) NEAR/3 (arrest* OR resuscitation)) OR asystole*):ab,ti,kw) AND ((extracorporeal* OR ECMO OR ECLS OR "cardiopulmonary bypass"):ab,ti,kw).

Embase 30 September 2020

Equation:

('hypothermia'/exp OR 'warming'/exp OR (rewarming OR hypothermi* OR (body NEAR/3 temperature NEAR/3 change*)):ab,ti,kw) AND ('heart arrest'/exp OR (((heart OR cardiac OR cardiopulmonary) NEAR/3 (arrest* OR resuscitation)) OR asystole*):ab,ti,kw) AND ('extracorporeal circulation'/exp OR 'extracorporeal oxygenation'/exp OR (extracorporeal* OR ECMO OR ECLS OR "cardiopulmonary bypass"):ab,ti,kw) NOT ('animal'/exp NOT 'human'/exp).

Web of Science 30 September 2020

Equation:

TS = ((rewarming OR hypothermi* OR (body NEAR/3 temperature NEAR/3 change*)) AND (((heart OR cardiac OR cardiopulmonary) NEAR/3 (arrest* OR resuscitation)) OR asystole*) AND (extracorporeal* OR ECMO OR ECLS OR "cardiopulmonary bypass")).

Table S3. Source and characteristics of the 70 selected patients identified through the literature review (n = 64 references).

Source	First Author	Patients (70)
1	Gilbert M.	1
2	Coleman E.	1
3	Carlsen A. W.	1
4	Darocha T.	1
5	Meyer M.	1
6	Sansone F	2
7	Simek M.	1
8	Adhikari S. P.	1
9	Bolgiano E.	1
10	Boue Y.	1
11	Carlsen A. W.	2
12	Cohen D. J.	1
13	Deiml R.	1
14	Eckert I.	1
15	Eich C.	1
16	Elbers P. W.G.	1
17	Fister M.	1
18	Forti A.	1
19	Graeff I.	1
20	Holmström P.	1
21	Hungerer S.	1
22	Husby P.	1
23	Incagnoli P.	1
24	Lund F. K.	1
25	Maeder M. B.	1
26	Eckhard M.	1
27	Marquis C.	1
28	McCormack J.	1
29	Merz S.	1
30	Morley D.	1
31	Mulpur A. K.	1
32	Mutschlechnera H.	1
33	Niehaus M. T.	2
34	Oberhammer R.	1
35	Romlin B. S.	1
36	Rünitz K	1
37	Svendsen O. S.	1
38	Thalmann M.	1
39	Tirilomis A.	1
40	Waters D. J.	1
41	Wik L.	1
42	Dobson J.	1
43	Kakizaki R.	1
44	Mair P.	1
45	Mayor Pleines A.-F.	1
46	Mosesso V. N. Jr.	1

47	Norberg W. J.	1
48	Beaton C.	1
49	Kosinski S.	1
50	Riera J.	1
51	Boué Y.	2
52	Heller K.	1
53	Gretenkort P.	2
54	Köpcke V. J.	1
55	Umlauf V. N.	1
56	Bellanova G.	1
57	Nesemann M. E.	1
58	Shephard R. J.	1
59	Brat R.	1
60	Husby P.	1
61	Cooper S. S.	1
62	Antretter H.	1
63	Wickstrom P.	2
64	Cha S.	1

Complete list of the 64 references identified through the literature review.

1. Gilbert M, Busund R, Skagseth A, Nilsen PA, Solbø JP. Resuscitation from accidental hypothermia of 13.7 degrees C with circulatory arrest. Lancet. 2000 Jan 29;355(9201):375-6. doi: 10.1016/S0140-6736(00)01021-7. PMID: 10665559.
2. Coleman E, Doddakula K, Meeke R, Marshall C, Jahangir S, Hinchion J. An atypical case of successful resuscitation of an accidental profound hypothermia patient, occurring in a temperate climate. Perfusion. 2010 Mar;25(2):103-6. doi: 10.1177/0267659110366066. Epub 2010 Mar 23. PMID: 20332176.
3. Carlsen AW, Winnerkvist AM, Greiff G. A 95 year-old suffering circulatory arrest after accidental hypothermia: a case report. BMC Geriatr. 2017 Oct 26;17(1):249. doi: 10.1186/s12877-017-0646-6. PMID: 29070019; PMCID: PMC5657076.
4. Darocha T, Kosinski S, Moskwa M, Jarosz A, Sobczyk D, Galazkowski R, Slowik M, Drwila R. The Role of Hypothermia Coordinator: A Case of Hypothermic Cardiac Arrest Treated with ECMO. High Alt Med Biol. 2015 Dec;16(4):352-5. doi: 10.1089/ham.2015.0055. Epub 2015 Aug 24. PMID: 26301723; PMCID: PMC4685480.
5. Meyer M, Pelurson N, Khabiri E, Siegenthaler N, Walpoth BH. Sequela-free long-term survival of a 65-year-old woman after 8 hours and 40 minutes of cardiac arrest from deep accidental hypothermia. J Thorac Cardiovasc Surg. 2014 Jan;147(1):e1-2. doi: 10.1016/j.jtcvs.2013.08.085. Epub 2013 Oct 28. PMID: 24176273.
6. Sansone F, Flocco R, Zingarelli E, Dato GM, Punta G, Parisi F, Forsennati PG, Bardi GL, Imbastaro I, Chiolero C, Balossino A, Borin P, Peretto V, del Ponte S, Casabona R. Hypothermic cardiac arrest in the homeless: what can we do? J Extra Corpor Technol. 2011 Dec;43(4):252-7. PMID: 22416606; PMCID: PMC4557429.
7. Simek M, Hajek R, Bruk V, Fabikova K, Nemec P, Raimr J, Husar R, Hubacek P. Accidental deep hypothermia with cardiac arrest. Prompt complete recovery after rewarming by extracorporeal circulation. Case report. Biomed Pap Med Fac Univ Palacky Olomouc Czech Repub. 2007 Jun;151(1):95-7. doi: 10.5507/bp.2007.017. PMID: 17690748.
8. Adhikari SP, Lowenstein RA. Severe hypothermia complicated by home heating fuel oil contamination. Prehosp Emerg Care. 2005 Oct-Dec;9(4):454-6. doi: 10.1080/10903120500255180. PMID: 16263682.
9. Bolgiano E, Sykes L, Barish RA, Zickler R, Eastridge B. Accidental hypothermia with cardiac arrest: recovery following rewarming by cardiopulmonary bypass. J Emerg Med. 1992 Jul-Aug;10(4):427-33. doi: 10.1016/0736-4679(92)90271-t. PMID: 1430979.
10. Boue Y, Lavolaine J, Bouzat P, Matraxia S, Chavanon O, Payen JF. Neurologic recovery from profound accidental hypothermia after 5 hours of cardiopulmonary resuscitation. Crit Care Med. 2014 Feb;42(2):e167-70. doi: 10.1097/CCM.0b013e3182a643bc. PMID: 24158171.
11. Carlsen AW, Skjaervold NK, Berg NJ, Karlsen Ø, Gunnarson E, Wahba A. Swedish-Norwegian co-operation in the treatment of three hypothermia victims: a case report. Scand J Trauma Resusc Emerg Med. 2017 Jul 17;25(1):73. doi: 10.1186/s13049-017-0418-5. PMID: 28716150; PMCID: PMC5514463.
12. Cohen DJ, Cline JR, Lepinski SM, Bowman HM, Ireland K. Resuscitation of the hypothermic patient. Am J Emerg Med. 1988 Sep;6(5):475-8. doi: 10.1016/0735-6757(88)90251-3. PMID: 3415744.

13. Deiml R, Hess W. Erfolgreiche Therapie eines Kreislaufstillstands bei akzidenteller Hypothermie durch die Extrakorporale Zirkulation [Successful therapy of a cardiac arrest during accidental hypothermia using extracorporeal circulation]. Anaesthesist. 1992 Feb;41(2):93-8. German. PMID: 1562099.
14. Eckert I, Imboden P, Paal P, Koppenberg J. Good neurological outcome after accidental hyopthermia presenting with asytole. Anaesthesist. 2017 Mar;66(3):186-188. English. doi: 10.1007/s00101-017-0271-y. Epub 2017 Feb 7. Erratum in: Anaesthesist. 2017 Apr;66(4):293-294. PMID: 28175939.
15. Eich C, Bräuer A, Kettler D. Recovery of a hypothermic drowned child after resuscitation with cardiopulmonary bypass followed by prolonged extracorporeal membrane oxygenation. Resuscitation. 2005 Oct;67(1):145-8. doi: 10.1016/j.resuscitation.2005.05.002. PMID: 16129537.
16. Elbers PW, Craenen AJ, Driessen A, Stehouwer MC, Munsterman L, Prins M, van Iterson M, Bruins P, Ince C. Imaging the human microcirculation during cardiopulmonary resuscitation in a hypothermic victim of submersion trauma. Resuscitation. 2010 Jan;81(1):123-5. doi: 10.1016/j.resuscitation.2009.09.032. Epub 2009 Dec 5. PMID: 19963312.
17. Fister M, Knafelj R, Radsel P, Zlicar M, Goslar T, Noc M. Cardiopulmonary Resuscitation with Extracorporeal Membrane Oxygenation in a Patient with Profound Accidental Hypothermia and Refractory Ventricular Fibrillation. Ther Hypothermia Temp Manag. 2019 Mar;9(1):86-89. doi: 10.1089/ther.2018.0011. Epub 2018 Aug 10. PMID: 30096259.
18. Forti A, Brugnaro P, Rauch S, Crucitti M, Brugger H, Cipollotti G, Strapazzon G. Hypothermic Cardiac Arrest With Full Neurologic Recovery After Approximately Nine Hours of Cardiopulmonary Resuscitation: Management and Possible Complications. Ann Emerg Med. 2019 Jan;73(1):52-57. doi: 10.1016/j.annemergmed.2018.09.018. Epub 2018 Nov 9. PMID: 30420231.
19. Graeff I, Schacher S, Lenkeit S, Widmann CN, Schewe JC. Beyond the limits - ECPR in putative fatal circumstances. CJEM. 2018 Oct;20(S2):S70-S73. doi: 10.1017/cem.2018.32. Epub 2018 Mar 25. PMID: 29573754.
20. Holmström P, Boyd J, Sorsa M, Kuisma M. A case of hypothermic cardiac arrest treated with an external chest compression device (LUCAS) during transport to re-warming. Resuscitation. 2005 Oct;67(1):139-41. doi: 10.1016/j.resuscitation.2005.04.013. PMID: 16129539.
21. Hungerer, Sven & Ebenhoch, Michael & Geiser, T. & Bühren, Volker. (2013). Akzidentelle, tiefe Hypothermie in der Bergrettung. Notfall & Rettungsmedizin. 16. 114. 10.1007/s10049-012-1659-5.
22. Husby P, Andersen KS, Owen-Falkenberg A, Steien E, Solheim J. Accidental hypothermia with cardiac arrest: complete recovery after prolonged resuscitation and rewarming by extracorporeal circulation. Intensive Care Med. 1990;16(1):69-72. doi: 10.1007/BF01706328. PMID: 2312909.
23. Incagnoli P, Bourgeois B, Teboul A, Laborie JM. Survie sans séquelles d'un arrêt cardiaque avec hypothermie sévère à 22 degrés C: importance de la stratégie de prise en charge préhospitalière [Resuscitation from accidental hypothermia of 22 degrees C with circulatory arrest: importance of prehospital management]. Ann Fr Anesth Reanim. 2006 May;25(5):535-8. French. doi: 10.1016/j.annfar.2006.01.011. Epub 2006 Mar 3. PMID: 16516435.
24. Lund FK, Torgersen JG, Flaatten HK. Heart rate monitored hypothermia and drowning in a 48-year-old man. survival without sequelae: a case report. Cases J. 2009 Aug 18;2:6204. doi: 10.4076/1757-1626-2-6204. PMID: 19918562; PMCID: PMC2769272.
25. Maeder MB, Lischke V, Berner A, Reisten O, Pietsch U, Pasquier M. A patient with polytrauma, hypothermia and cardiac arrest after delayed mountain rescue. CMAJ. 2018 Oct 22;190(42):E1263. doi: 10.1503/cmaj.70338. PMID: 30348744; PMCID: PMC6199164.
26. Mark E, Jacobsen O, Kjerstad A, Naesheim T, Busund R, Bahar R, Jensen JK, Skorpen PK, Bjertnaes LJ. Hypothermic cardiac arrest far away from the center providing rewarming with extracorporeal circulation. Int J Emerg Med. 2012 Feb 1;5:7. doi: 10.1186/1865-1380-5-7. PMID: 22296952; PMCID: PMC3296662.
27. High frequency oscillatory ventilation associated with cardiopulmonary bypass in the management of drowning and deep hypothermia. Marquis C., Journois D., Teboul A., Bellenfant F., Safran D. JEUR (2005) 18:2 (100-101). Date of Publication: Jun 2005
28. McCormack J, Percival D. HEMS advanced trauma team retrieval of a patient with accidental hypothermic cardiac arrest for ECMO therapy. Resuscitation. 2016 Aug;105:e23. doi: 10.1016/j.resuscitation.2016.05.013. Epub 2016 May 30. PMID: 27255955.
29. Merz S, Kumle B, Simon M, Benk C, Henschen M. Near drowning of a 1aEuroyear-old boy. Care from first responder to extracorporeal measures. Springer, 2017 Dec; 20(8);682-687, DOI: 10.1007/s10049-017-0301-y
30. Morley D, Yamane K, O'Malley R, Cavarocchi NC, Hirose H. Rewarming for accidental hypothermia in an urban medical center using extracorporeal membrane oxygenation. Am J Case Rep. 2013;14:6-9. doi: 10.12659/AJCR.883728. Epub 2013 Jan 8. PMID: 23569552; PMCID: PMC3614333.
31. Mulpur AK, Mirsadraee S, Hassan TB, McKeague H, Kaul P. Refractory ventricular fibrillation in accidental hypothermia: salvage with cardiopulmonary bypass. Perfusion. 2004;19(5):311-4. doi: 10.1191/0267659104pf760oa. PMID: 15506037.

32. Mutschlechner H, Lorenz I, Oberhammer R, Brugger H, Paal P. Hyperinsulinaemia may impair outcome after hypothermic cardiac arrest. Resuscitation. 2009 Aug;80(8):959. doi: 10.1016/j.resuscitation.2009.04.034. Epub 2009 May 29. PMID: 19481850.
33. Niehaus MT, Pechulis RM, Wu JK, Frei S, Hong JJ, Sandhu RS, Greenberg MR. Extracorporeal Membrane Oxygenation (ECMO) for Hypothermic Cardiac Deterioration: A Case Series. Prehosp Disaster Med. 2016 Oct;31(5):570-1. doi: 10.1017/S1049023X16000637. Epub 2016 Aug 5. PMID: 27492653.
34. Oberhammer R, Beikircher W, Hörmann C, Lorenz I, Pycha R, Adler-Kastner L, Brugger H. Full recovery of an avalanche victim with profound hypothermia and prolonged cardiac arrest treated by extracorporeal re-warming. Resuscitation. 2008 Mar;76(3):474-80. doi: 10.1016/j.resuscitation.2007.09.004. Epub 2007 Nov 7. PMID: 17988783.
35. Romlin BS, Winberg H, Janson M, Nilsson B, Björk K, Jeppsson A, Drake G, Claesson A. Excellent Outcome With Extracorporeal Membrane Oxygenation After Accidental Profound Hypothermia (13.8°C) and Drowning. Crit Care Med. 2015 Nov;43(11):e521-5. doi: 10.1097/CCM.0000000000001283. PMID: 26317568.
36. Rünitz K, Thornberg K, Wanscher M. Dybt hypoterm og multitraumatiseret kvinde genoplivet efter langvarigt hjertestop [Resuscitation of severely hypothermic and multitraumatised female following long-term cardiac arrest]. Ugeskr Laeger. 2009 Jan 26;171(5):328-9. Danish. PMID: 19176170.
37. Svendsen ØS, Grong K, Husby P. Neuroprotective treatment strategies after rewarming from accidental hypothermia. Resuscitation. 2018 Jan;122:e9-e10. doi: 10.1016/j.resuscitation.2017.10.017. Epub 2017 Oct 24. PMID: 29079509.
38. Thalmann M, Trampitsch E, Haberfellner N, Eisendle E, Kraschl R, Kobinia G. Resuscitation in near drowning with extracorporeal membrane oxygenation. Ann Thorac Surg. 2001 Aug;72(2):607-8. doi: 10.1016/s0003-4975(00)02307-9. PMID: 11515909.
39. Tirilomis A, Friedrich M, Braeuer A, Heise D, Bireta C, Steinmetz M, Ruschewski W. Drowning Accident with Deep Hypothermia. Luciani GB, Ündar A. Welcome to the 11th International Conference on Pediatric Mechanical Circulatory Support Systems and Pediatric Cardiopulmonary Perfusion. Artif Organs. 2015 May;39(5):397-9. doi: 10.1111/aor.12522. PMID: 25953234.
40. Waters DJ, Belz M, Lawse D, Ulstad D. Portable cardiopulmonary bypass: resuscitation from prolonged ice-water submersion and asystole. Ann Thorac Surg. 1994 Apr;57(4):1018-9. doi: 10.1016/0003-4975(94)90229-1. PMID: 8166500.
41. Wik L, Kiil S. Use of an automatic mechanical chest compression device (LUCAS) as a bridge to establishing cardiopulmonary bypass for a patient with hypothermic cardiac arrest. Resuscitation. 2005 Sep;66(3):391-4. doi: 10.1016/j.resuscitation.2005.03.011. PMID: 15992987.
42. Dobson JA, Burgess JJ. Resuscitation of severe hypothermia by extracorporeal rewarming in a child. J Trauma. 1996 Mar;40(3):483-5. doi: 10.1097/00005373-199603000-00032. PMID: 8601876.
43. Kakizaki R, Bunya N, Uemura S, Odagiri A, Kasai T, Narimatsu E. Takotsubo cardiomyopathy developed during rewarming of accidental hypothermia with extracorporeal membrane oxygenation. Acute Med Surg. 2019 Mar 1;6(2):201-205. doi: 10.1002/ams2.399. PMID: 30976450; PMCID: PMC6442525.
44. Mair P, Schwarz B, Kornberger E, Balogh D. Case 5-1997. Successful resuscitation of a patient with severe accidental hypothermia and prolonged cardiocirculatory arrest using cardiopulmonary bypass. J Cardiothorac Vasc Anesth. 1997 Dec;11(7):901-4. doi: 10.1016/s1053-0770(97)90131-2. PMID: 9412895.
45. Mayor Pleines AF, Guyot E, Yersin B. Hypothermie accidentelle: un cas extreme de reanimation avec succès [Accidental hypothermia: an extreme case of successful resuscitation]. Praxis (Bern 1994). 2006 Jul 5;95(27-28):1075-9. German. doi: 10.1024/0369-8394.95.27.1075. PMID: 16888924.
46. Mosesso VN Jr, Liebman J. Back from the brink. Emergency cardiopulmonary bypass creates potential for saving the nearly dead. JEMS. 2014 Dec;39(12):50-3, 57. PMID: 25630184.
47. Norberg WJ, Agnew RF, Brunsvold R, Sivanna P, Browdie DA, Fisher D. Successful resuscitation of a cold water submersion victim with the use of cardiopulmonary bypass. Crit Care Med. 1992 Sep;20(9):1355-7. doi: 10.1097/00003246-199209000-00026. PMID: 1521451.
48. Beaton, C., Hanson, J., & Tsang, J. (2020). Survival after accidental hypothermia and cardiac arrest using emergency department-initiated extracorporeal membrane oxygenation. CJEM, 22(5), 726-728. doi:10.1017/cem.2020.381
49. Kosiński S, Drzewiecka A, Pasquier M, Gołba KS, Podsiadło P, Drwiła R, Darocha T. Successful Defibrillation at a Core Temperature of 18.2 Degrees Celsius. Wilderness Environ Med. 2020 Jun;31(2):230-234. doi: 10.1016/j.wem.2020.01.003. Epub 2020 Apr 21. PMID: 32331951.
50. Riera J, Argudo E, Ruiz-Rodríguez JC, Rodríguez-Lecoq R, Ferrer R. Full neurological recovery 6 h after cardiac arrest due to accidental hypothermia. Lancet. 2020 May 16;395(10236):e89. doi: 10.1016/S0140-6736(20)30751-0. PMID: 32416783.
51. Boué Y, Payen JF, Torres JP, Blancher M, Bouzat P. Full neurologic recovery after prolonged avalanche burial and cardiac arrest. High Alt Med Biol. 2014 Dec;15(4):522-3. doi: 10.1089/ham.2014.1082. PMID: 25313580.
52. Heller K, Salata S. Cardiopulmonary arrest after cold water immersion and hypothermia. J Emerg Nurs. 1988 Jan-Feb;14(1):5-8. PMID: 3279252.

53. Ice-water drowning with cardiac arrest: Is resuscitation by means of extracorporeal circulation realistic? (Eiswasser-ertrinken mit herz-kreislauf-stillstand: Ist eine wiederbelebung unter einsatz der extra-korporalen zirkulation realistisch?) Gretenkort P., Weissenfels M. Notarzt (2000) 16:4 (133-137). Date of Publication: 2000
54. Köpcke J, Westphal B, Benad G. Erfolgreiche Reanimation eines unterkühlten Patienten mittels Extrakorporaler Zirkulation--Ein Fallbericht [Successful resuscitation of a hypothermic patient with extracorporeal circulation--a case report]. Anaesthesiol Reanim. 1996;21(6):159-62. German. PMID: 9090950.
55. ECMO in drowning pediatric patients. Life-saving option or efforts in vain? ECMO nach Ertrinkungsunfällen im Kindesalter: Lebensrettende Option oder vergebliche Mühe? Umlauf V.N., Bierbach B., Ksellmann A. Intensiv- und Notfallbehandlung (2018) 43:1 (11-19). Date of Publication: 2018
56. Bellanova G, Motta A, Mazzetti C, Motter M, Fabris L, DeVigili G, Liguori G. Damage Control Strategy and aggressive resuscitation in polytraumatized patient with severe hypothermia. Importance of multidisciplinary management from the territory to the operating room. Case report. Ann Ital Chir. 2013 Jul-Aug;84(4):445-9. PMID: 23917428.
57. Nesemann ME, Busch HM Jr, Gundersen AL, Gundersen AE, Newcomer KL. Asystolic cardiac arrest in hypothermia. Wis Med J. 1983 Jan;82(1):19-20. PMID: 6338660.
58. Shephard RJ. Asphyxial death of a young skier. J Sports Med Phys Fitness. 1996 Sep;36(3):223-7. PMID: 8979653.
59. Brát R, Suk M, Bárta J, Schichel T, Kozák D, Kucera T, Prusenovský P, Urbanec R. Resuscitace hluboce podchlazeného nemocného s pouzitím mimotělního oběhu [Resuscitation of a patient with deep hypothermia using extracorporeal circulation]. Rozhl Chir. 2002 Jun;81(6):279-81. Czech. PMID: 12149869.
60. Husby P, Steien E, Andersen KS, Solheim J. Dyp aksidentell hypotermi med asystoli. Vellykket behandling med hjerte-lunge-maskin etter langvarig kardiopulmonal resuscitering [Deep accidental hypothermia with asystole. A successful treatment with heart-lung machine after prolonged cardiopulmonary resuscitation]. Tidsskr Nor Laegeforen. 1991 Jan 20;111(2):183-5. Norwegian. PMID: 1998175.
61. Cooper SS, Papadimos TJ, Campbell JA, Cerilli GJ, Omer S, Braida AL, Hassan AM. Successful resuscitation of an elderly man with deep accidental hypothermia using portable extracorporeal circulation in the emergency department: a case report. J Med Case Rep. 2008 May 9;2:150. doi: 10.1186/1752-1947-2-150. PMID: 18471286; PMCID: PMC2396176.
62. Antretter H, Dapunt OE, Mueller LC. Portable cardiopulmonary bypass: resuscitation from prolonged ice-water submersion and asystole. Ann Thorac Surg. 1994 Dec;58(6):1786-7. doi: 10.1016/0003-4975(94)91698-5. PMID: 7979764.
63. Wickstrom P, Ruiz E, Lija GP, Hinterkopf JP, Haglin JJ. Accidental hypothermia: core rewarming with partial bypass. Am J Surg. 1976 May;131(5):622-5. doi: 10.1016/0002-9610(76)90029-5. PMID: 5902.
64. Cha SO, Axelsson B, Hallbäck DA, Bomfim V. Accidentell hypotermi. Två fallbeskrivningar [Accidental hypothermia. 2 case reports]. Lakartidningen. 1993 Jun 2;90(22):2157-8. Swedish. PMID: 8502073.
